# Plasma extracellular vesicles bearing PD-L1, CD40, CD40L or TNF-RII are significantly reduced after treatment of AIDS-NHL

**DOI:** 10.1038/s41598-022-13101-8

**Published:** 2022-06-02

**Authors:** Laura E. Martínez, Shelly Lensing, Di Chang, Larry I. Magpantay, Ronald Mitsuyasu, Richard F. Ambinder, Joseph A. Sparano, Otoniel Martínez-Maza, Marta Epeldegui

**Affiliations:** 1 UCLA AIDS Institute and David Geffen School of Medicine, University of California, Los Angeles, Biomedical Sciences Research Building Room 173, Los Angeles, CA 90095 USA; 2 Department of Obstetrics and Gynecology, David Geffen School of Medicine, University of California, Los Angeles, CA USA; 3 Department of Biostatistics, University of Arkansas for Medical Sciences, Little Rock, AR USA; 4 Division of Hematologic Malignancies, Johns Hopkins Sidney Kimmel Comprehensive Cancer Center, John Hopkins University, Baltimore, MD USA; 5 Division of Hematology Oncology, Mount Sinai, NY USA; 6 Jonsson Comprehensive Cancer Center, University of California, Los Angeles, CA USA

**Keywords:** Predictive markers, HIV infections, Non-hodgkin lymphoma

## Abstract

Emerging evidence shows that tumor cells secrete extracellular vesicles (EVs) that carry bioactive cell surface markers, such as programmed death-ligand 1 (PD-L1), which can modulate immune responses and inhibit anti-tumor responses, potentially playing a role in lymphomagenesis and in promoting the growth of these cancers. In this study, we investigated the role of EVs expressing cell surface molecules associated with B cell activation and immune regulation. We measured levels of EVs derived from plasma from 57 subjects with AIDS-related non-Hodgkin lymphoma (AIDS-NHL) enrolled in the AIDS Malignancies Consortium (AMC) 034 clinical trial at baseline and post-treatment with rituximab plus concurrent infusional EPOCH chemotherapy. We found that plasma levels of EVs expressing PD-L1, CD40, CD40L or TNF-RII were significantly reduced after cancer treatment. AIDS-NHL patients with the diffuse large B cell lymphoma (DLBCL) tumor subtype had decreased plasma levels of EVs bearing PD-L1, compared to those with Burkitt’s lymphoma. CD40, CD40L and TNF-RII-expressing EVs showed a significant positive correlation with plasma levels of IL-10, CXCL13, sCD25, sTNF-RII and IL-18. Our results suggest that patients with AIDS-NHL have higher levels of EVs expressing PD-L1, CD40, CD40L or TNF-RII in circulation before cancer treatment and that levels of these EVs are associated with levels of biomarkers of microbial translocation and inflammation.

## Introduction

Chronic HIV-1 infection increases the risk of B cell non-Hodgkin lymphoma (NHL). Although combination anti-retroviral therapy (cART) has improved the overall survival of persons living with HIV-1 infection^1–8^, non-Hodgkin lymphoma (NHL) remains a significant cause of morbidity and mortality among HIV-1-infected individuals in the post-cART era^9,10^. Anti-CD20 or rituximab (monoclonal anti-CD20 antibody) immunotherapy has been shown to significantly improve the outcome of patients with HIV-associated CD20-positive lymphomas^11^. CD20 is a cell surface tetraspan receptor expressed on most B cells, which plays a significant role in the differentiation, maturation, and activation of B cells, and most B cell NHLs are positive for CD20. Several studies have shown that rituximab, in combination with an infusional regimen of etoposide, vincristine, doxorubicin, cyclophosphamide, and prednisone (EPOCH) chemotherapy, is highly effective in HIV-associated B cell NHL^12–14^. The association between HIV+ status and survival in NHL appears to be attenuated after controlling for more aggressive presentation and lower frequency of rituximab use in HIV+ individuals^15^.

We previously showed that treatment of patients with AIDS-associated NHL (AIDS-NHL) enrolled in the AIDS Malignancy Consortium (AMC) 034 trial (AMC-034), which involved treatment with rituximab plus infusional EPOCH chemotherapy, resulted in decreased plasma levels of several B cell activation-associated molecules, such as IL-6, IL-10, CXCL13, sCD27, and sCD30, where reduced plasma levels persisted for one year after the completion of treatment^16^. Pre-treatment levels of CXCL13, IL-6, and IL-10 were also associated with response to therapy, as well as overall survival^16^. In addition, subjects who responded to therapy had overall lower pre-treatment levels of sCD14, sCD25, IL-18 and sTNF-RII, and higher pre-treatment levels of EndoCab IgM^17^.

We have also shown that CD19^+^ B cells expressing PD-L1 are significantly elevated in HIV+ subjects 1–4 years prior to AIDS-NHL diagnosis^18^. Most PD-L1-expressing cells had a CD19^+^CD24^++^CD38^++^ phenotype characteristic of B-regulatory cells, which secrete inhibitory cytokines such as IL-10, to suppress adaptive immune responses^18^. Therapeutic antibodies can block PD-L1’s interaction with PD-1 and reactivate the anti-tumor immune response^19^. Several studies have already used tumor PD-L1 as a predictive biomarker for clinical response to anti-PD-1 therapy^20–22^. Moreover, PD-L1-expressing B cells that secrete extracellular vesicles bearing PD-L1 may play an important role in the etiology and pathogenesis of AIDS-NHL.

Extracellular vesicles (EVs) are lipid-bound membrane vesicles released by most cells into the extracellular milieu that carry bioactive molecules, including proteins, lipids, metabolites, DNA, mRNA, and microRNA, and can express immunoregulatory molecules on their surface, and like cytokines and chemokines, can circulate in peripheral blood to distant sites^23^. Tumor cell-derived EVs participate in the formation and progression of different cancer processes, such as remodeling of the tumor microenvironment, angiogenesis, metastasis, and in some cases, drug-resistance, which remains one the biggest challenges in cancer therapy^24^. Thus, EVs are promising non-invasive biomarkers that may correlate with tumor progression and/or response to therapy, and prognosis for different types of cancer^24–26^.

PD-L1-expressing EVs, which can be found in both peripheral blood circulation and the tumor microenvironment, can inhibit anti-tumor immune responses and promote tumor-mediated disease progression^27–31^. Studies have shown that PD-L1^+^-exosomes are significantly higher in melanoma patients who do not respond to anti-PD-1 therapy^32^. Moreover, PD-L1-bearing exosomes are as efficient at inhibiting T cell activation as melanoma cancer cells^33^. Exosomal PD-L1 levels have been shown to be significantly higher in non-small cell lung cancer (NSCLC) patients with advanced tumor stage, larger tumor size, and distant metastasis^34^.

In this study, we performed a prospective analysis to investigate whether extracellular vesicles isolated from plasma of patients with AIDS-NHL at baseline, and post-treatment, express molecules associated with B cell activation and immune signaling, such as PD-L1, CD40, CD40L, B7-H3, TNF-RII or IL-6Rα. We sought to determine if EVs bearing these molecules correlated with biomarkers known to be significantly decreased after AIDS-NHL treatment.

## Results

### Characterization of plasma-derived EVs from AIDS-NHL patients

**Figure 1:**
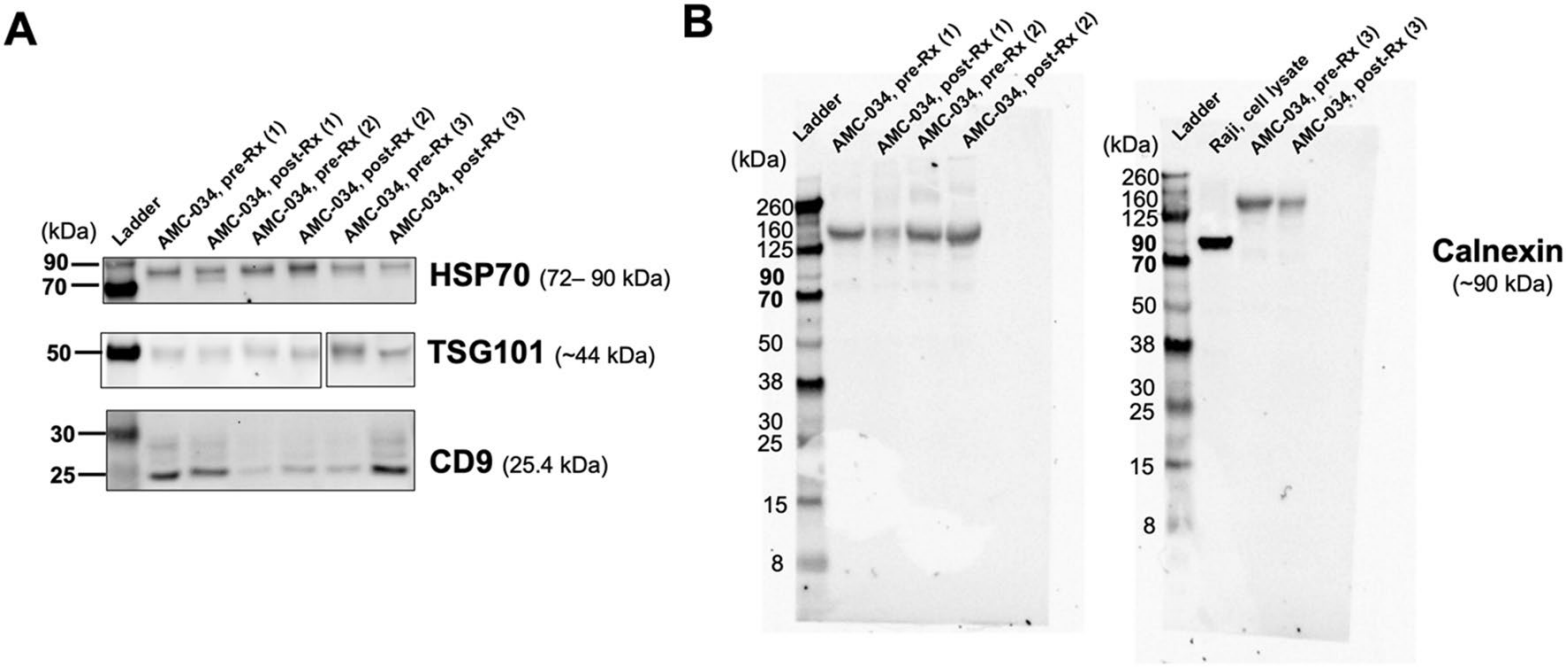
Characterization of extracellular vesicles isolated from plasma of AMC-034 trial participants at pre-treatment (baseline) and post-treatment. (**A**) Western blot analysis of EVs demonstrating the presence of exosome-specific markers, such as the tetraspanin CD9, TSG101, and HSP70, and (**B**) a non-exosomal maker, Calnexin (endoplasmic reticulum marker). 20 µg was used from each sample. Results are from plasma-derived EVs of matched pre-treatment (pre-RX or baseline) (N = 3) and post-treatment (post-Rx) (N = 3) plasma samples of AMC-034 trial subjects, including protein from the lysate of the Raji cell line (20 µg). The exposure time for the HSP70 blot was 90 s. For the TSG101 blot, boxed lines delineate that AMC pre- and post-Rx sample 3 was run in a separate gel, and blots were exposed for 30 s each. The exposure time for the CD9 blot was 120 s. Full blots are shown for Calnexin, where AMC pre- and post-Rx sample 3 was run in a separate gel along with the cell lysate from the Raji cell line. Each blot was exposed for 30 s.

Extracellular vesicles were isolated from plasma of 57 AMC-034 clinical trial participants at pre-treatment (baseline) and post-treatment (with rituximab and concurrent infusional EPOCH chemotherapy), using a total exosome isolation precipitation reagent (Invitrogen). Two subjects did not have post-treatment samples. Western blot analysis showed that EVs expressed proteins known to be enriched in exosomes, such as the tetraspanin CD9, TSG101, and HSP70 (Fig. 1A), confirming that these preparations were enriched for EVs. Calnexin was used as the non-exosomal marker and it was only detected in protein from lysate of Raji cells, a Burkitt’s lymphoma cell line (Fig. 1B), but was not detected in EVs isolated from the plasma of AIDS-NHL patients.

### AIDS-NHL patients showed decreased plasma levels of EVs bearing molecules associated with immune activation following cancer treatment

**Figure 2:**
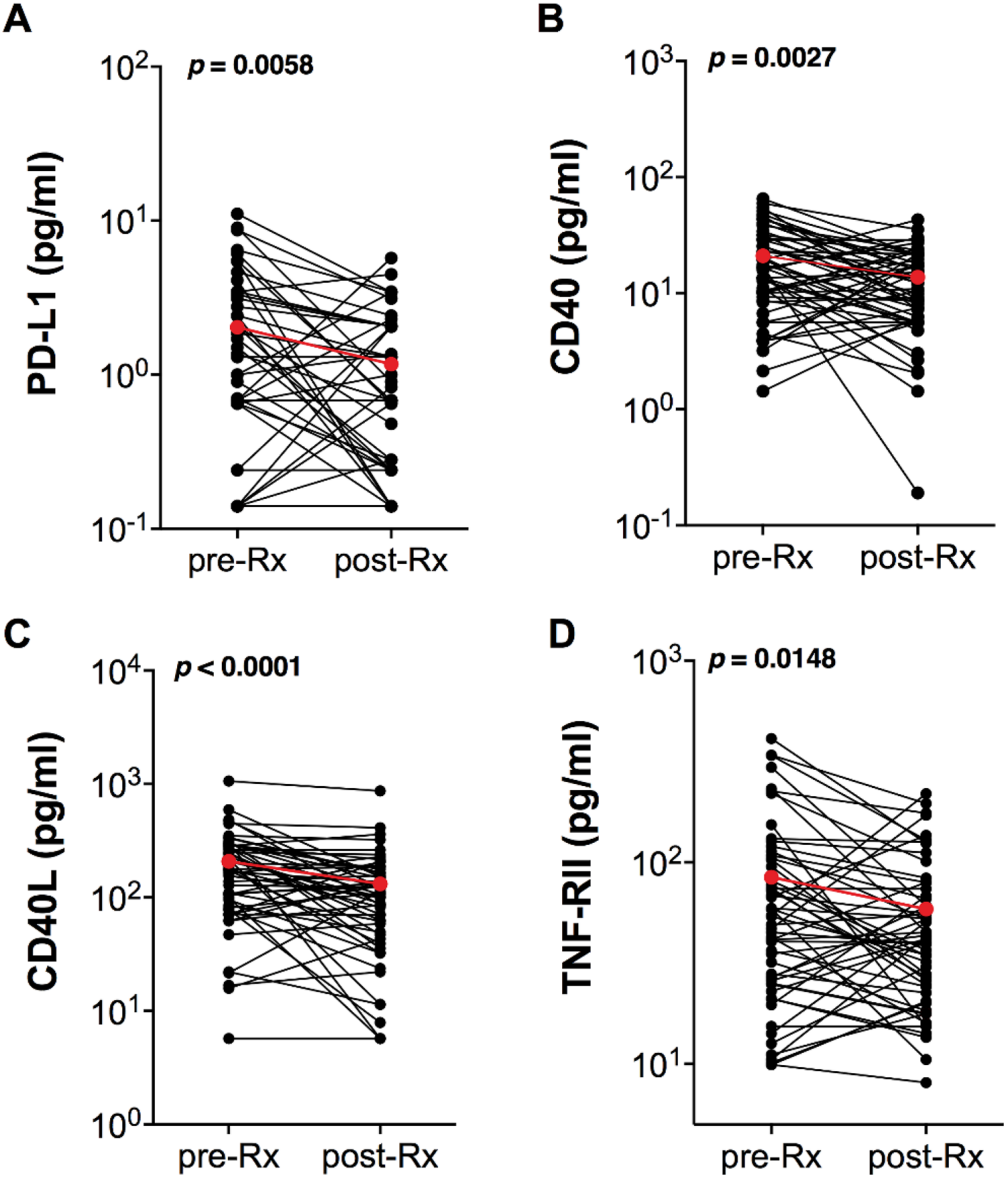
Plasma-derived extracellular vesicles expressing PD-L1, TNF-RII, CD40 or CD40L were significantly decreased after cancer treatment. Levels of exosomal PD-L1 (pg/ml) (**A**); CD40 (**B**), CD40L (**C**), and TNF-RII (**D**) isolated from plasma of AMC-034 trial subjects compared at pre-treatment (baseline) (pre-Rx) (N = 55) and post-treatment (post-Rx) (N = 55), as determined by Luminex multiplex immunometric assay. Each black circle corresponds to each patient and their mean value at pre- and post-treatment is provided (mean values were calculated from duplicate wells). Mean values of pre-Rx and post-Rx are shown as red circles and lines (PD-L1, mean pre-Rx (2.03 pg/ml) and post-Rx (1.17 pg/ml); CD40, mean pre-Rx (20.99 pg/ml) and post-Rx (13.73 pg/ml); CD40L mean pre-Rx (207.90 pg/ml) and post-Rx (131.82 pg/ml); and TNF-RII mean pre-Rx (84.26 pg/ml) and post-Rx (58.58 pg/ml). Statistical comparisons were made using two-sample non-parametric Wilcoxon rank sum tests.

We then evaluated whether EVs expressed cell surface markers on their surfaces that are important in immune regulatory responses and that play a role in lymphomagenesis. Thus, we measured plasma levels of EVs expressing molecules important for B and T cell signaling (CD40 and CD40L), molecules with immune modulatory activities (PD-L1, B7 immune checkpoint molecule B7-H3 [CD276], and ICAM-1 [CD54)]), TNF-RII, the receptor for TNFα and an indicator of clinical response to treatment and survival in AIDS-NHL^16,17^; IL-6Rα, which is part of the IL-6 receptor, and a molecule involved in the regulation of cell death, Fas Ligand (FasL). We measured the expression of these cell surface markers on EVs (PD-L1, CD40, CD40L, B7-H3, TNF-RII, IL-6Rα, ICAM-1 or FasL) using a multiplexed bead beased immunometric assay (Luminex). AIDS-NHL patients showed decreased plasma levels of EVs bearing PD-L1 (*p* = 0.006), CD40 (p = 0.003), CD40L (p < 0.001) or TNF-RII (p = 0.015) after cancer treatment, compared to baseline plasma levels (Wilcoxon signed rank test) (Fig. 2A–D). The data presented in Fig. 2 is for 55 patients at pre- and post-treatment. For results on PD-L1-bearing EVs, we found that 25 of 55 patients had higher PD-L1 values at pre-treatment compared to its corresponding, matched post-treatment value; 11 of 55 patients had higher values at post-treatment compared to pre-treatment; and 19 of 55 patients had values that were the same at pre- and post-treatment. For data on CD40-bearing EVs, 35 of 55 patients had higher CD40 values at pre- compared to post-treatment; 18 of 55 patients had higher values at post- compared to pre-treatment, and 2 of 55 patients had values that were the same at pre- and post-treatment. For data on CD40L-bearing EVs, 37 of 55 patients had higher CD40L values at pre- compared to post-treatment; 16 of 55 patients had higher values at post- compared to pre-treatment, and 2 of 55 patients had values that were the same at pre- and post-treatment. For data on TNF-RII-bearing EVs, 35 of 55 patients had higher TNF-RII values at pre- compared to post-treatment; 18 of 55 patients had higher values at post- compared to pre-treatment, and 2 of 55 patients had values that were the same at pre- and post-treatment.

### Plasma-derived EVs expressing PD-L1 were significantly lower in AIDS-NHL patients with the DLBCL tumor subtype

**Figure 3:**
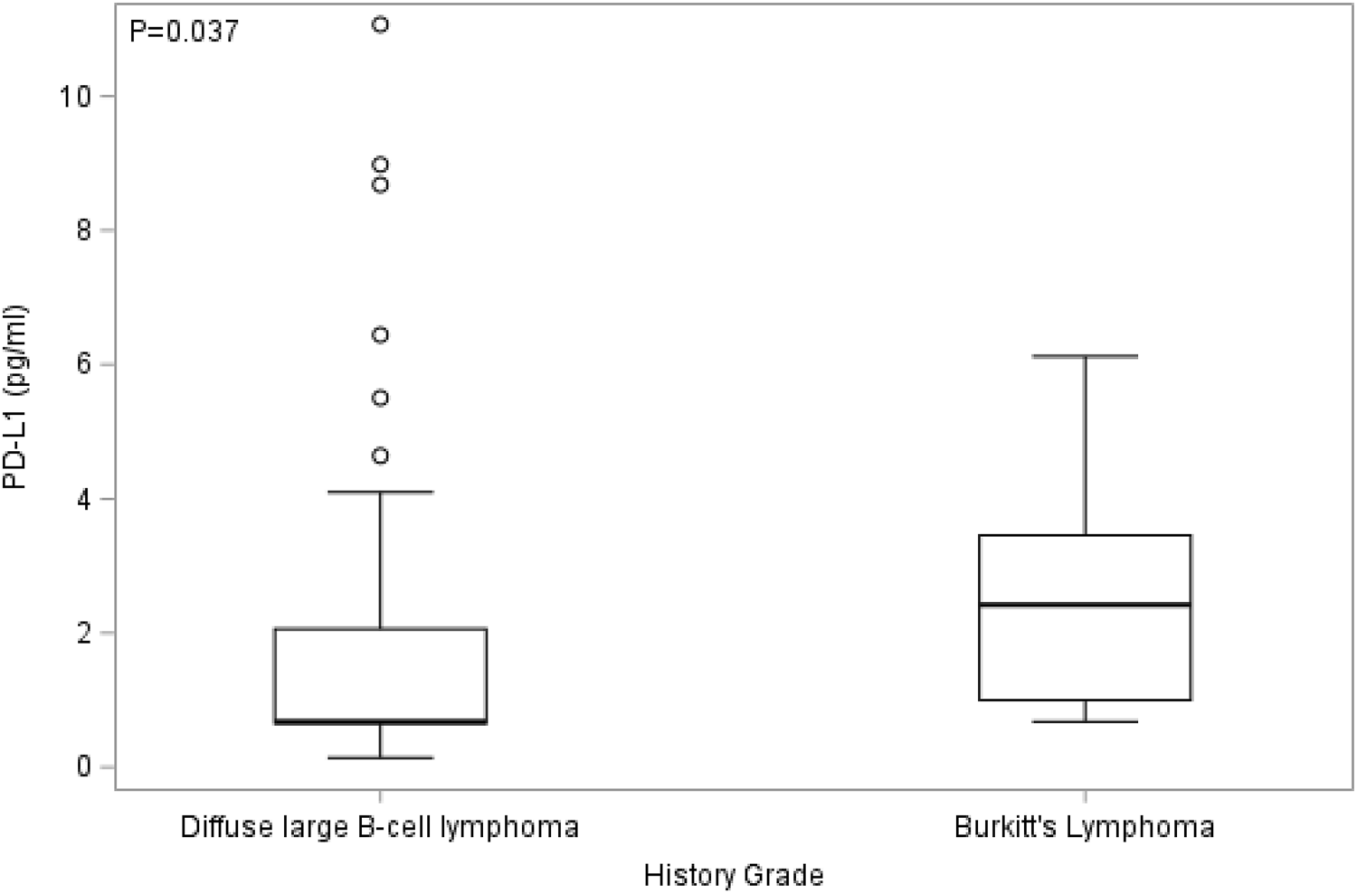
Plasma levels of PD-L1-expressing EVs were significantly lower at baseline in AMC-034 trial participants with diffuse large B cell lymphoma (DLBCL) than in those with Burkitt lymphoma. Box plots showing the distributions of PD-L1-expressing EVs at baseline for DLBCL (N = 46; Median score = 0.7) and Burkitt’s lymphoma (BL) (N = 7; Median score = 2.4) tumor subtypes among AMC-034 trial subjects at baseline. Wilcoxon two-sample tests were conducted.

**Figure 4:**
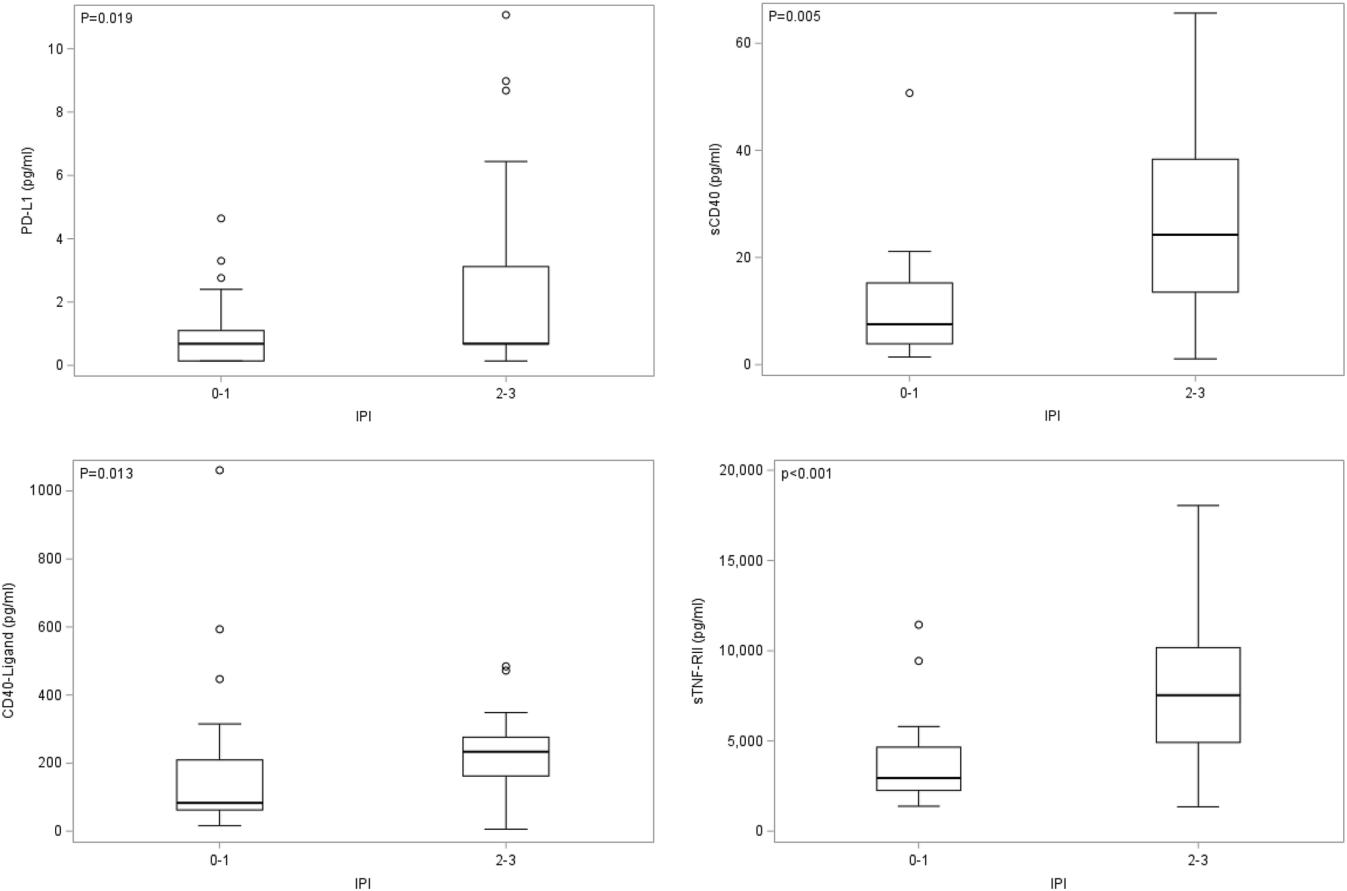
AMC-034 trial participants with DLBCL with IPI scores of 2 -3 showed increased expression of EVs bearing PD-L1, CD40, CD40L or TNF-RII at baseline. Comparison of EVs bearing PD-L1 (**A**), CD40 (**B**), CD40L (**C**), or TNF-RII (**D**) according to IPI scores 0–1 (n = 20) and 2–3 (n = 26) for DLBCL tumor subtype. Box plots show the distribution of EVs expressing these markers; Wilcoxon two-sample tests were conducted.

We then evaluated any associations between blood circulating EVs expressing the different immune cell surface markers (PD-L1, CD40, CD40L, B7-H3, TNF-RII, IL-6Rα, ICAM-1 or FasL) and tumor subtype of AIDS-NHL patients. AIDS-NHL patients who had the DLBCL tumor subtype showed significantly reduced plasma levels of PD-L1-expressing EVs when compared to patients with Burkitt’s lymphoma tumor subtype (*p* = 0.037, Wilcoxon two-sample test) (Fig. 3). We further compared EVs according to IPI score for AIDS-NHL patients with DLBCL tumor subtype, and found that the group with IPI scores between 2–3 had significantly higher EVs bearing PD-L1, CD40, CD40L or TNF-RII compared to the group with an IPI score between 0 and 1 (Fig. 4).

### Plasma-derived EVs bearing different immune markers correlated with biomarkers known to be significantly associated with AIDS-NHL risk and prognosis

We then investigated whether EVs expressing the different immune cell surface markers (PD-L1, CD40, CD40L, B7-H3, TNF-RII, IL-6Rα, ICAM-1 or FasL) correlated with biomarkers previously shown to be associated with AIDS-NHL risk^16^. We did not observe that any of the cell surface markers in EVs had prognostic value, however the sample size is small for survival analyses and the comparison was not a priori powered. In prior studies, we measured plasma levels of EndoCab IgM by ELISA, and quantified plasma levels of molecules associated with B cell stimulation/activation (IL-6, sCD23, sCD25, sCD30, CXCL13, sTNFRII, sCD44, IL-10), macrophage activation (BAFF/BLyS, IL-18, CCL2/MCP-1, TNFα, TARC/CCL17, sCD163), and microbial translocation (sCD14, LBP) by Luminex multiplex immunometric assay, as described in Martínez et al.^17^. Here, we determined if a correlation between plasma-derived EVs and plasma biomarker levels existed prior to cancer treatment. We found that EVs bearing PD-L1 significantly correlated with plasma levels of IL-10 at baseline for the subset with available IL-10 data (n = 41 of 57 total subjects, Spearman’s ρ = 0.38, *p* = 0.014). Furthermore, we found a significant association between EVs bearing PD-L1 and plasma levels of IL-10 at baseline for subjects with DLBCL tumor subtype (n = 32, Spearman’s ρ = 0.47, *p* = 0.007). We did not find any correlations for PD-L1-expressing EVs with HIV viral load, CD4^+^ T cell count, or any of the biomarkers examined at baseline (data not shown). However, CD40-expressing EVs significantly correlated with HIV viral load (Spearman’s ρ = 0.40, *p* = 0.003), as well as EVs bearing TNF-RII (Spearman’s ρ = 0.59, *p* < 0.001) or IL-6Rα (Spearman’s ρ = 0.38, *p* = 0.005) (Table 1). None of the plasma-derived EVs correlated with CD4^+^ T cell count (data not shown). Moreover, CD40-expressing EVs positively correlated with plasma levels of IL-10, CXCL13, sCD25, sTNF-RII, IL-18, and sCD163, while CD40L-expressing EVs correlated with plasma levels of IL-10, CXCL13, sCD25, sTNF-RII, IL-18, sCD163, and CCL17 at baseline (Table 1). TNF-RII-expressing EVs correlated with plasma levels of IL-6, IL-10, CXCL13, sCD25, sTNF-RII, IL-18, BAFF, sCD14, sCD44, and CXCL10; and IL-6Rα-expressing EVs correlated with plasma levels of IL-10, CXCL13, sCD25, sTNF-RII, IL-18, sCD163, BAFF, and CXCL10 at baseline (Table 1).

**Table 1:** Correlations for plasma-derived extracellular vesicles expressing CD40, CD40L, TNF-RII or IL-6Rα with HIV viral load and biomarkers of AIDS-NHL risk at baseline (n = 41–57).

	CD40^+^ EVs	CD40L^+^ EVs	TNF-RII^+^ EVs	IL-6Rα^+^ EVs
**HIV viral load**				
Spearman’s ρ^a^	0.40	0.16	0.59	0.38
*p*^b^	0.003	0.254	< 0.001	0.005
**IL-6**				
Spearman’s ρ	0.28	0.17	0.42	0.28
*p*	0.075	0.293	0.006	0.080
**IL-10**				
Spearman’s ρ	0.43	0.45	0.44	0.43
*p*	0.005	0.003	0.004	0.005
**CXCL13**				
Spearman’s ρ	0.42	0.37	0.64	0.53
*p*	0.007	0.018	< 0.001	< 0.001
**sCD25**				
Spearman’s ρ	0.38	0.34	0.51	0.38
*p*	0.004	0.011	< 0.001	0.004
**sTNF-RII**				
Spearman’s ρ	0.40	0.35	0.68	0.43
*p*	0.002	0.008	< 0.001	0.001
**IL-18**				
Spearman’s ρ	0.35	0.27	0.48	0.44
*p*	0.008	0.042	< 0.001	0.001
**sCD163**				
Spearman’s ρ	0.28	0.28	0.24	0.43
*p*	0.038	0.038	0.074	0.001
**CCL17**				
Spearman’s ρ	0.05	0.29	− 0.03	0.10
*p*	0.729	0.030	0.819	0.445
**BAFF**				
Spearman’s ρ	0.18	0.20	0.47	0.31
*p*	0.173	0.128	< 0.001	0.018
**sCD14**				
Spearman’s ρ	0.22	0.15	0.42	0.17
*p*	0.099	0.252	0.001	0.194
**sCD44**				
Spearman’s ρ	0.20	0.09	0.30	0.20
*p*	0.129	0.494	0.024	0.126
**CXCL10**				
Spearman’s ρ	0.13	0.19	0.48	0.31
*p*	0.343	0.160	< 0.001	0.018

The number of subjects with available HIV viral load data was n = 52; the number of subjects with available IL-6, IL-10, and CXCL13 data was n = 41; and the number of subjects with available data for all other biomarkers was n = 57.

^a^Spearman’s ρ (rho) represents the correlation coefficient.

^b^*p*: *p*-value testing if correlation is significantly different from 0.

When we examined EVs bearing B7-H3, we found significant negative correlations with plasma levels of CXCL10 (Spearman’s ρ = − 0.27, *p* = 0.042), sCD44 (Spearman’s ρ = − 0.39, *p* = 0.003), and EndoCab IgM (Spearman’s ρ = − 0.26, *p* = 0.048) at baseline. EVs bearing FasL correlated with plasma levels of sCD44 (Spearman’s ρ = 0.32, *p* = 0.015), CXCL10 (Spearman’s ρ = 0.33, *p* = 0.013), and TNF-α (Spearman’s ρ = 0.35, *p* = 0.008) at baseline. Significant associations were found for ICAM-1-expressing EVs and plasma levels of BAFF (Spearman’s ρ = 0.27, *p* = 0.042), sCD163 (Spearman’s ρ = 0.33, *p* = 0.012), and IL-18 (Spearman’s ρ = 0.30, *p* = 0.023) at baseline.

### Characterizing associations among plasma-derived EVs expressing the different immune cell surface molecules

Multiple immune activation and immuneregulatory molecules can be enriched on the surface of circulating extracellular vesicles. Thus, we further investigated whether any strength and/or direct associations existed among the plasma-derived EVs and determined Spearman’s correlation coefficients for each comparison. We found that plasma levels of PD-L1-expressing EVs directly correlated with plasma levels of EVs expressing CD40, CD40L, TNF-RII, IL-6Rα, ICAM-1, or FasL (Table 2). A significant, indirect correlation was found between PD-L1 and B7-H3-expressing EVs (Table 2). CD40-expressing EVs had significant correlations with all the other EVs examined, with the exception of B7-H3 expressing EVs (Table 2). CD40L-expressing EVs showed significant correlations with all other EVs, with the exception of B7-H3 or FasL expressing EVs (Table 2). Moreover, TNF-RII-expressing EVs significantly correlated with all the EVs examined, while IL-6Rα correlated with all other EVs but not with B7-H3 or FasL expressing EVs (Table 2). ICAM-1-expressing EVs correlated with all other EVs but not with B7-H3. Lastly, FasL-expressing EVs correlated with all other EVs but not with EVs expressing CD40L or IL-6Rα (Table 2). These results suggest that some EVs share extracellular surface molecules on their surface.

**Table 2:** Correlation between plasma-derived EVs expressing PD-L1, CD40, CD40L, TNF-RII or IL-6Rα at baseline (n = 57).

	PD-L1^+^ EVs	CD40^+^ EVs	CD40L^+^ EVs	TNF-RII^+^ EVs	IL-6Rα^+^ EVs
**PD-L1**^**+**^** EVs**					
Spearman’s ρ^a^	1.00	0.55	0.38	0.49	0.33
*p*^b^		< 0.001	0.003	0.0001	0.011
**CD40**^**+**^** EVs**					
Spearman’s ρ	0.55	1.00	0.60	0.66	0.49
*p*	< 0.001		< 0.001	< 0.001	0.001
**CD40L**^**+**^** EVs**					
Spearman’s ρ	0.38	0.60	1.00	0.48	0.58
*p*	0.003	< 0.001		< 0.001	< 0.001
**TNF-RII**^**+**^** EVs**					
Spearman’s ρ	0.49	0.66	0.48	1.00	0.68
*p*	< 0.001	< 0.001	< 0.001		< 0.001
**IL6Rα**^**+**^** EVs**					
Spearman’s ρ	0.33	0.49	0.58	0.68	1.00
*p*	0.011	< 0.001	< 0.001	< 0.001	
**FasL**^**+**^** EVs**					
Spearman’s ρ	0.40	0.27	0.22	0.34	0.12
*p*	0.002	0.040	0.094	0.011	0.359
**B7-H3**^**+**^** EVs**					
Spearman’s ρ	− 0.33	− 0.18	− 0.10	− 0.37	− 0.09
*p*	0.011	0.169	0.473	0.005	0.484
**ICAM**^**+**^** EVs**					
Spearman’s ρ	0.47	0.56	0.31	0.53	0.54
*p*	< 0.001	< 0.001	0.019	< 0.001	< 0.001

^a^Spearman’s ρ (rho) represents the correlation coefficient.

^b^*p*: *p*-value testing if correlation is significantly different from 0.

## Discussion

Extracellular vesicles released from cancer cells can function as messengers to regulate other cells in the tumor microenvironment, and contribute to tumor growth and tumor progression^35^. PD-L1 is highly expressed in tumor cells and can be secreted in EVs to alter the immune system response in the tumor microenvironment^25^. In this study, we measured plasma concentrations of PD-L1-expressing EVs and compared those with clinical and pathologic features of patients with AIDS-NHL who were taking part in the AMC-034 trial, such as HIV viral load, CD4^+^ T cell count, tumor subtype, response to cancer treatment, patient outcome measures, and evaluated associations with plasma levels of molecules important for AIDS-NHL risk^16,17^.

We found that plasma levels of PD-L1-expressing EVs were significantly higher in AIDS-NHL patients prior to the initiation of rituximab and EPOCH chemotherapy. A significant correlation between plasma levels of EVs expressing PD-L1 and plasma levels of IL-10 was observed at baseline. It is important to note that DLBCL tumor subtype had overall reduced plasma levels of PD-L1^+^ EVs compared to the Burkitt’s NHL subgroup, raising the possibility that the positive and significant association between PD-L1^+^ EVs and plasma levels of IL-10 is due to tumor type. Although this is difficult to acertain, since there were only seven patients with Burkitt’s NHL, we did see that the correlation was stronger in the DLBCL subset compared to both tumor types combined. These results suggest that plasma levels of IL-10 at baseline may act as a negative immunomodulatory factor to inhibit immune responses associated with circulation of PD-L1^+^ EVs. We previously showed that pre-treatment levels of IL-10 were associated with response to therapy, as well as overall survival, in AIDS-NHL^16^. Thus, plasma levels of PD-L1-expressing EVs and IL-10 hold promise as effective prognostic biomarkers in AIDS-NHL.

We then examined other molecules important for B and T cell signaling, such as CD40, CD40L, and B7-H3 (CD276), which have co-stimulatory/co-inhibitory immunoregulatory functions^36^, and are highly expressed in different types of cancers^37^. We also evaluated the expression of TNF-RII on EVs, whose expression has been linked to tolerogenic immune reactions, including a subset of T-regulatory cells^38^ and IL-10-producing B cells^39^; and EVs expressing IL-6Rα, which is of high interest since our previous work has shown that IL-6 has prognostic value as an indicator of subsequent response to AIDS-NHL treatment and survival^16^. Moreover, we also quantified plasma levels of FasL-expressing EVs. FasL can be expressed in both membrane bound and soluble forms and has been shown to be secreted in vesicles from activated T cells to induce Fas-mediated apoptosis^40^.

We found that EVs bearing CD40 correlated with plasma levels of IL-10, CXCL13, sCD25, sTNF-RII, IL-18, and sCD163, while TNF-RII-expressing EVs correlated with IL-6, IL-10, CXCL13, sCD25, sTNF-RII, IL-18, BAFF, sCD14, sCD44, and CXCL10 at baseline. IL-6Rα-expressing EVs correlated with several plasma biomarkers, such as IL-10, CXCL13, sCD25, sTNF-RII, IL-18, sCD163, BAFF, and CXCL10 at baseline. Thus, we have found that molecules important for B cell signaling and that promote B cell survival are enriched in blood circulating EVs at baseline in AIDS-NHL patients, and that these plasma-derived EVs correlate with biomarkers of AIDS-NHL risk. CD40, TNF-RII or IL-6Rα on EVs may facilitate communication between lymphoma cells and distant immune cells of the tumor microenvironment. AIDS-NHL patients also had increased plasma levels of EVs bearing CD40L, which may also facilitate communication and/or the recruitment of tumor-infiltrating B lymphocytes. Moreover, we did not find significant differences in plasma levels of EVs bearing FasL at baseline and post-treatment, but we did find correlations between FasL-expressing EVs and plasma levels of biomarkers for AIDS-NHL risk (sCD44, CXCL10, and TNF-α) at baseline, suggesting that they can mediate tumor immune responses and/or tumor immune escape. Morever, the expression of these different immune cell surface molecules on plasma-derived EVs can mediate early events in lymphomagenesis and play a vital role in cancer metastasis, further interacting with different immune cells in the tumor microenvironment and modulating immune responses. EVs expressing PD-L1 can modulate T cell responses, promoting the growth of tumor cells and/or inhibiting T cells in distant draining lymph nodes^41^.

These results suggest that EVs may serve as potential biomarkers for prospective immunotherapy-focused clinical trials for AIDS-NHL prior to, and after, initiating therapy. Further studies will include the proteomic profiling of plasma-derived EVs from AIDS-NHL patients in order to define other immunoregulatory molecules associated with immune activation and/or dysfunction, as well as the cytokine profiling of EVs. Extracellular vesicle cargos are heterogeneous and mirror the landscape of EV producing cells. Therefore, it will be important to profile the repertoire of EV proteomes, which can be subjected to imaging mass cytometry to examine EVs in the tumor microenvironment.

Various studies have shown that HIV-1-infected cells secrete exosomes that carry viral RNA and proteins and that can be transported to other cells in the microenvironment^42–46^. These EVs have the potential to have myriad effects on the pathogenesis of AIDS-NHL and disease progression. Thus, it will be important to elucidate the molecular mechanisms mediated by plasma-derived EVs to determine if EVs bearing PD-L1 and/or other B7-molecules have immunosuppressive properties that may inhibit T cell activation and/or anti-tumor responses.

Circulating tumor-derived EVs are being examined for the early detection of a variety of cancers, which have the potential to discover new chemopreventive strategies and/or anti-cancer drug therapies. Our study supports that EVs are associated with biomarkers contributing to chronic B cell activation, macrophage activation, and microbial translocation, all factors that are associated with AIDS-NHL risk and pathogenesis. These findings have relevance to HIV-infected individuals with high levels of tumor cells secreting EVs in blood circulation while undergoing treatment with anti-B-cell therapy drugs, including rituximab and chemotherapy agents.

## Methods

### AIDS Malignancies Consortium (AMC) 034 study population

Of the 106 AIDS-NHL patients enrolled in an AIDS Malignancy Consortium (AMC) trial, AMC protocol #034 (AMC-034), which compared infusional combination chemotherapy (EPOCH: etoposide, vincristine, doxorubicin, cyclophosphamide, and prednisone) with concurrent or sequential rituximab^16^, plasma specimens were available from 57 patients with intermediate- or high-grade HIV-associated B cell NHL (50 patients had DLBCL, 17 had Burkitt’s lymphoma, and 2 were classified only as lymphoma). Clinical responses were defined as described in the report detailing AMC-034 trial results, including clinical characteristics of patients^16^. Briefly, the median age of lymphoma patients was 42.6 ± 8.8 years. Lymphoma patients had a median HIV-1 plasma level of 9,908 [inter-quartile range (IQR) between 492.5 and 45,660] and a median CD4^+^ T cell number of 187 cells/mm^3^ [IQR between 82 and 333]. Plasma was collected before the initiation of therapy at the end of the first cycle (within a week or less of treatment), and at 6 months and one year following the completion of treatment.

### Extracellular vesicle isolation from plasma of AMC-034 trial subjects

Plasma samples of 0.2 ml were differentially centrifuged at 2000×*g* for 20 min at room temperature to remove cell debris. The supernatant of clarified plasma was then transferred to a new tube and centrifuged at 10,000×*g* for 20 min at room temperature to further remove any remaining debris. The supernatant containing the clarified plasma was then transferred to a new tube with 0.5 ml volume of 1× PBS and mixed thoroughly, and 0.2 ml volume of the exosome precipitation reagent (from plasma) was added to the sample (Total Exosome Isolation (Plasma) Kit, Invitrogen, Cat No:4484450). The plasma/reagent solution was mixed by vortexing until the solution was homogenous and then the samples were incubated at room temperature for 10 min. After the incubation period, samples were centrifuged at 10,000×*g* for 5 min at room temperature to pellet exosomes. The supernatant was retrieved by pipet and discarded. The EV pellet was centrifuged for an additional 30 s at 10,000×*g* to collect any residual reagent. The residual supernatant was carefully discarded with a pipet. Then, 300 μl of 1× PBS was added to the pellet, resuspended with a pipet, and then lightly vortexed. Isolated EVs were stored at 4 °C or kept at − 20 °C until ready for downstream analysis, such as for Luminex multiplex immunometric assay and western blot analysis.

### Extracellular vesicle protein quantification and western blot analysis

Protein quantification of plasma-derived EV preparations was performed by Micro BCA assay (Micro BCA Protein Assay Kit, Thermo Fisher Scientific). Absorbance was read at 562 nm using a Molecular Devices VersaMax microplate reader and data was analyzed by SoftMax Pro software (5.4) (Molecular Devices). For preparing samples for western blot analysis, EVs were lysed by adding an equal volume of 1X RIPA Lysis Buffer (pH 7.4 ± 0.1) (Santa Cruz Biotechnologies) with Protease Inhibitor Cocktail in DMSO (Santa Cruz Biotechnologies), 200 mM PMSF solution (in DMSO) (Santa Cruz Biotechnologies), and 100 mM Sodium Orthovanadate solution (in water) (Santa Cruz Biotechnologies), followed by incubation at 4 °C for 15 min. Preparations were normalized for protein content and 20 µg were prepared with 4× Protein Sample Loading Buffer (LI-COR) for a final concentration of 1×, and then incubated at 70 °C for 10 min. Samples were then loaded into each well of a pre-cast 4–12% Bis–Tris Mini Protein Gel (1.0 mm) (3.5–260 kd) (Invitrogen), run at 150 V, and then electrophoretic transferred to an Millipore Immobilin^®^-FL PVDF Membrane (0.45 µm) using a Mini-Protean Tetra System (BioRad) at 100 V for 1 h. Membranes were immersed for 30s in 100% methanol then and wet in 1X Intercept^®^ TBS Blocking Buffer (1× TBS) (LI-COR) for 5 min. Membranes were then blocked with 1× TBS for 1 h at room temperature with gentle shaking, according to manufacturer’s instructions. Membranes were then incubated with the following primary antibodies: mouse monoclonal anti-CD9 (Developmental Studies Hybridoma Bank, Cat #602.29 cl.11) at 1:50; rabbit polyclonal anti-HSP70/HSPA1A (R&D Systems, Cat# AF1663) at 1:1000; mouse monoclonal anti-calnexin (Santa Cruz Biotechnology, Cat# sc-23954 at 1:1,000; or rabbit polyclonal anti-TSG101 (Proteintech, Cat#28283-1-AP) at 1:2000. All primary antibodies were diluted in 1× TBS with 0.2% Tween 20, and incubated overnight at 4 °C with gentle shaking. The next day, the blot was rinsed 3–5 times with 1× TBS-0.1% Tween 20 for 5 min each wash over a platform shaker, and then incubated with appropriate secondary antibodies: IRDye 800CW goat anti-mouse secondary antibody (1:10,000) (for detection of CD9 and Calnexin), IRDye 680RD goat anti-rabbit secondary antibody (1:10,000) (for detection of TSG101) or IRDye 800CW donkey anti-rabbit secondary antibody (1:10,000) (for detection of HSP70). Secondary antibodies were diluted in 1× TBS with 0.2% Tween 20 and 0.02% SDS for 1 h at room temperature with gentle shaking. The blot was then washed 3 times with 1× TBS-0.1% Tween 20 for 5 min each wash while shaking vigorously over a platform shaker at room temperature, and then finally rinsed in 1× TBS to remove residual Tween 20. Blots were then immediately scanned at 700 nm or 800 nm using a ChemiDoc Touch Imaging System (UCLA AIDS Institute) and images were collected.

### Quantification of plasma-derived EVs expressing PD-L1 and other immunoregulatory molecules by Luminex multiplex immunometric assay

EVs were retrieved from − 20 °C and left at 4 °C for thawing. Samples were treated with 0.1% Tween-20 in 1× PBS to inactivate HIV (final concentration of 0.05%) and then left at room temperature for at least 30 min before loading on to Luminex multiplex assay plates with custom-made panels produced by R&D Systems. Customized plates consisted of a panel of microparticles pre-coated with analyte-specific antibodies against PD-L1, CD40, CD40L, TNF-RII, IL-6Rα, B7-H3, ICAM-1 (CD54), and Fas Ligand (FasL). Exosomes isolated from plasma of AMC-034 patients were added to the plate in duplicate and incubated, followed by a biotin antibody and by a streptavidin–phycoerythrin conjugate. The fluorescence intensity of each analyte's microparticles was quantified using a BioPlex 200 (Luminex) System Analyzer (Bio-Rad, Hercules, California, USA), and the data analyzed using BioPlex Manager (v 4.1.1) software. A standard curve was generated for each biomarker. Standard curve values ranged from 900 to 1.23 pg/ml for PD-L1; 5430 to 7.45 pg/ml for CD40; 50,630 to 69.45 pg/ml for CD40L; 2680 to 3.68 pg/ml for TNF-RII; 25,320 to 34.73 pg/ml for IL-6Rα; 238,470 to 327.12 pg/ml for B7-H3; 2,040,400 to 2798.83 pg/ml for ICAM-1; and 2200 to 3.02 pg/ml for Fas Ligand. In some instances, extrapolated values were utilized, which are values that are based on a fluorescence signal that is above the background level of detectable fluorescence for the Luminex analyzer, but below the lowest value of the standard curve. For quality control, pre-treatment and post-treatment exosome samples were equally distributed across reaction plates, and duplicates were included across the reaction plates to calculate coefficients of variation. Laboratory personnel were blinded to the pre- and post-treatment status of samples.

To determine associations between EVs and biomarkers previously identified for AIDS-NHL risk, we used data previously acquired and reported in Ref.^17^. Briefly, plasma levels of EndoCab IgM (Hycult Biotech, Uden, The Netherlands) were determined by ELISA, according to the manufacturers’ instructions. Plasma levels of all other biomarkers (sCD14, LBP, FABP2, IL-18, CCL2/MCP-1, sCD163, IP-10/CXCL10, TARC/CCL17, TNF-α, BAFF/BLyS, sTNF-RII, sCD44, and sIL2Rα/sCD25) were determined using the Luminex multiplex assay platform with custom-made panels, as previously described^17^.

### Statistical analysis

Changes in plasma-derived EVs bearing PD-L1 were compared pre- to post-cancer treatment using paired nonparametric Wilcoxon sign-rank tests. In addition, pre-treatment (baseline) biomarkers were compared according to tumor type using the nonparametric Wilcoxon rank sum test. The relationships between PD-L1, CD40, CD40L, TNF-RII, IL-6Rα, B7-H3, ICAM-1 or FasL-expressing EVs and plasma levels of cytokines, chemokines, and prognostic biomarkers of AIDS-NHL were assessed using Spearman’s rank correlation coefficient. p values were not adjusted for multiple comparisons. In all cases, a two-tailed value of *p* < 0.05 was considered statistically significant.

### Informed consent and regulatory approval

The study was reviewed and approved by the Cancer Evaluation Therapy Program of the National Cancer Institute, and by the institutional review board at each participating institution. All patients provided written informed consent in accordance with the Declaration of Helsinki.

## Supplementary Information

Supplementary Figure 1.

## Data Availability

The datasets used and/or analyzed during the current study available from the corresponding author on reasonable request and AMC approval. Methods were performed in accordance to the relevant guidelines and regulation.
