# Supplementary material for: Plasma extracellular vesicles bearing PD-L1, CD40, CD40L or TNF-RII are significantly reduced after treatment of AIDS-NHL
Source: Sci Rep. 2022 Jun 2;12:9185. doi: 10.1038/s41598-022-13101-8 (PMC9163074; doi:10.1038/s41598-022-13101-8)
Supplement: Supplementary file 1 — Supplementary Figure 1. [file 41598_2022_13101_MOESM1_ESM.docx]

**
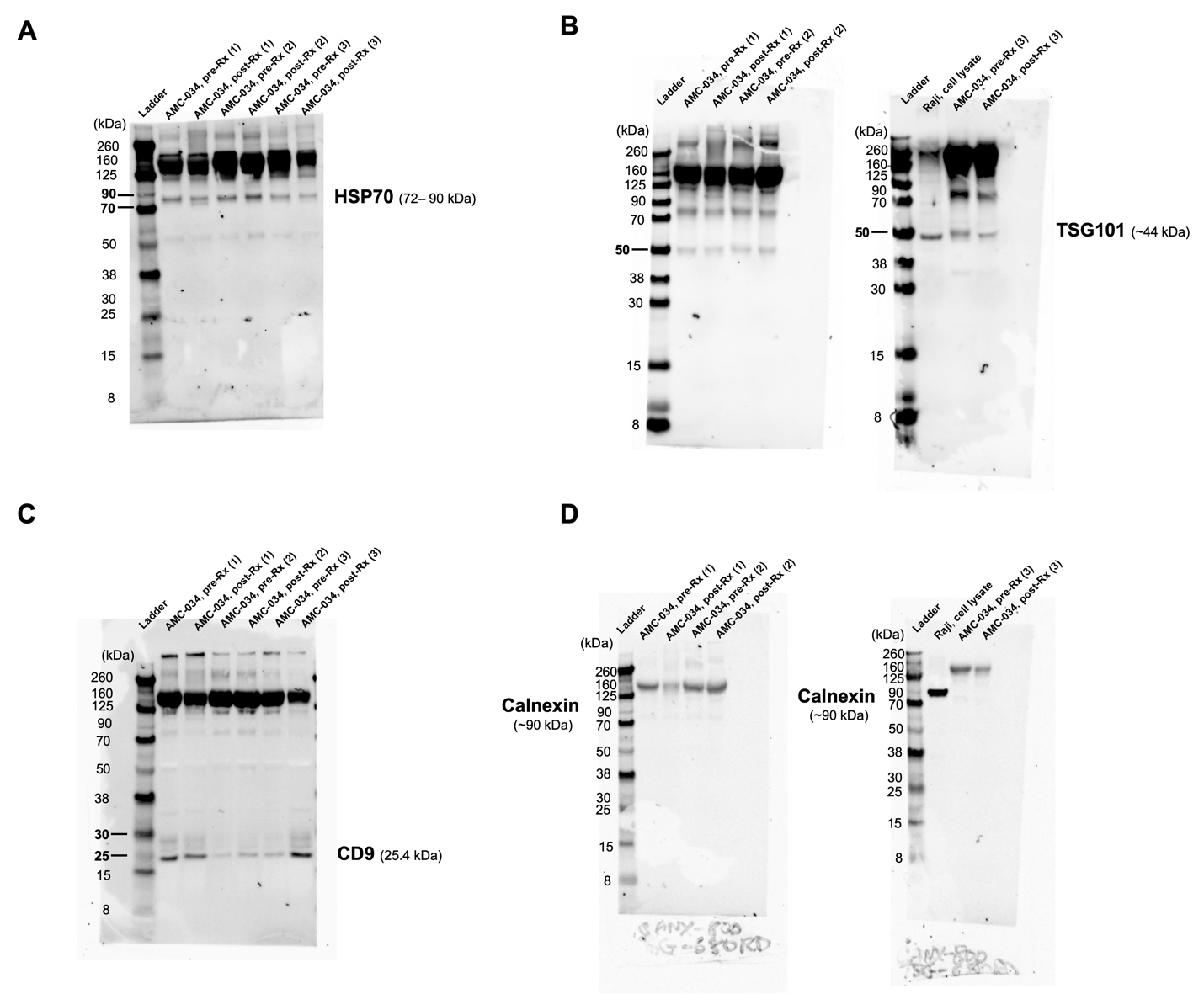
**

**Supplementary Figure 1. Full Western blot images of extracellular vesicles isolated from plasma of AMC-034 trial participants at pre-treatment (baseline) and post-treatment.** (A) Western blot analysis of EVs demonstrating the presence of HSP70 (A), TSG101 (B), CD9 (C), and Calnexin (D) in the Raji cell lysate. 20 µg was used from each sample. Results are from plasma-derived EVs of matched pre-treatment (pre-RX or baseline) (N=3) and post-treatment (post-Rx) (N=3) plasma samples of AMC-034 trial subjects. The exposure time for the HSP70 blot was 90 seconds. The exposure time for the CD9 blot was 120 seconds. For blots presented in (B) and (D), blots were multiplexed with primary antibodies against TSG101 (rabbit anti-human) and Calnexin (mouse anti-human) and with their respective secondary antibodies IRDye 680RD goat anti-rabbit secondary antibody and IRDye 800CW goat anti-mouse secondary antibody. For blots pertaining to TSG101 and Calnexin, AMC pre- and post-Rx sample 3 was run in a separate gel. Each blot was exposed for 30 seconds.
